# Examining factors that drive health-related students to seek help for psychological challenges

**DOI:** 10.5116/ijme.64a7.bffb

**Published:** 2023-07-24

**Authors:** Nidwaree Sojindamanee, Sirichai Hongsanguansri, Somboon Hataiyusuk, Nareemarn Neelapaichit, Karn Suttapanit, Ponjit Jithavech, Komsan Kiatrungrit

**Affiliations:** 1 Faculty of Medicine Ramathibodi Hospital, Faculty of Medicine Siriraj Hospital and National Institute for Child and Family Development, Mahidol University, Bangkok, Thailand; 2 Department of Psychiatry, Faculty of Medicine Ramathibodi Hospital, Mahidol University, Bangkok, Thailand; 3 Department of Psychiatry, Faculty of Medicine Siriraj Hospital, Mahidol University, Bangkok, Thailand; 4 Ramathibodi School of Nursing, Faculty of Medicine Ramathibodi Hospital, Mahidol University, Bangkok, Thailand; 5 Department of Emergency Medicine, Faculty of Medicine Ramathibodi Hospital, Mahidol University, Bangkok, Thailand; 6 Department of Communication Sciences and Disorders, Faculty of Medicine Ramathibodi Hospital, Mahidol University, Bangkok, Thailand

**Keywords:** Health-related student, seeking mental health care, mental health problems

## Abstract

**Objectives:**

This study aims to find the prevalence of mental health problems and the rates of seeking mental health care among health-related students, as well as identifying factors associated with seeking mental health care.

**Methods:**

A cross-sectional online survey was conducted among students from the Faculty of Medicine at a university in the 2021 academic year. A total of 832 students voluntarily completed an online survey measuring mental health problems and factors influencing mental health care seeking. Descriptive and analytic statistics including t-test, Pearson’s chi-square test, and binary logistic regression analysis, were used to analyze the data.

**Results:**

Among the participants, 46.80% (n = 389) reported experiencing mental health problems, but only 16.97% (n = 66) of them sought mental health care. Binary logistic regression analysis revealed that female (OR = 2.63 (1.08 - 6.43)) and LGBTQ (OR = 4.26 (1.36 – 13.37)) students, and those with a positive attitude toward professionals (OR = 1.10 (1.02 - 0.19)), were more likely to seek formal mental health care than those who did not.

**Conclusions:**

The study findings indicate that mental health problems are prevalent among health-related students, yet formal mental health care uptake is low. To address this issue, mental health screening programs, improved knowledge and attitudes about mental health care, and mental health training for academic staffs who work with health-related students are necessary. Future studies should explore interventions to increase the uptake of mental health services among health-related students.

## Introduction

The prevalence of mental health problems among college students is a serious issue with negative impacts on academic performance, relationships, and overall well-being. Mental health problems such as depression, anxiety, or self-harm are common among college students, and they can lead to disability or death if left untreated.^1.2^ Additionally, these problems waste valuable human and economic resources for treatment.^3^

College students may face various stressors, including competition in studying and exams, lack of leisure time, establishing new relationships, moving away from their home for studying,^4^ higher responsibility in their duties, and high expectations for their professions.^5^ These stressors can lead to mental health problems, particularly for health-related college students. The literature suggests that healthcare personnel are also at higher risk of experiencing anxiety, depression, and suicidal thoughts than the general population.^6^

Mental health problems are prevalent among college students, but only a small percentage seek help.^7^ A study in Thailand found that only 4.1% of people with a history of mental illness in the past year consulted with mental health specialists.^8^This is concerning, as untreated mental illness can lead to negative academic outcomes,^9^ a higher risk of subsequent mental health problems, low self-esteem, and an economic burden on society.^10^^, ^^11^ In addition, mental health problems that go untreated for a long time can become more severe and difficult to treat, potentially leading to suicide attempts.^12^ Therefore, it is crucial to understand the factors associated with seeking mental health care.

**Figure 1:**
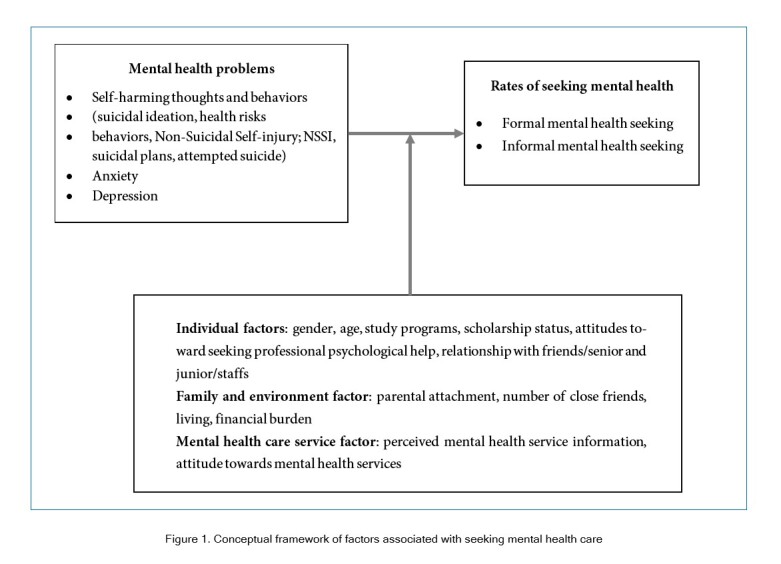
Conceptual framework of factors associated with seeking mental health care

The literature suggests that numerous factors can influence the decision to seek mental health care, with previous studies revealing that many students experiencing mental health issues do not seek help from family or friends. This reluctance may stem from relationship conflicts^13^ or a negative attitude towards stress and available services.^10^^, ^^14^^, ^^15^ Fear of stigmatization is another commonly cited factor, with research indicating that females may be more prone to this concern than males.^16^ Additionally, the quality of available mental health services has been identified as a key factor that can impact service utilization.^11^ To investigate the factors that impact students' decisions to seek mental health care, this study categorized variables into three major categories: individual, family and environmental, and mental health care services. The conceptual framework is depicted in Figure 1.

While previous studies have focused on specific fields or types of mental health problems, there is limited research on the rates of seeking mental health care among health-related students and factors associated with seeking mental health care. Thus, the aim of this study is to investigate the prevalence of mental health problems and the rates of seeking mental health care among college students, particularly health-related college students. Furthermore, this study aims to identify factors associated with seeking mental health care among them.

## Methods

### Study design and participants

This study was a cross-sectional survey of college students at a medical school in Bangkok. It was conducted during the 2021 academic year and included four bachelor’s programs: Doctor of Medicine (years 1-6), Nursing Science (years 1-4), Bachelor of Science in Communication Disorders (CSD) (years 1-4), and Bachelor of Science in Paramedicine (years 1-4). The sample size was calculated using the Taro Yamane formular (allowable error at 0.05), with a required sample size of at least 376 participants out of a total of 2,352 students. An additional 10% sample size was added to account for missing data.

There were 832 participants who responded to the questionnaire, consisting of 330 students in the Doctor of Medicine program (39.66%), 298 students in the Bachelor of Nursing program (35.82%), 98 students in the Bachelor of Science program in Communication Disorders (11.78%), and 106 students in the Bachelor of Science in Emergency Medicine Operations (12.74%). Among the participants, 52.64% (n = 438) were in their pre-clinical years.

Demographic information of the sample revealed that 18.27% were male (n = 152), 72.10% were female (n = 600), and 9.62% identified as LGBTQ (n = 80), with an average age of 19.97 years (min-max = 16-40, SD = 1.74). Most of the participants had a GPA of ≥ 3.50 (n= 447, 53.73%). In terms of financial status, 10.94% of the participants reported being financially burdened (n = 91), while 11.42% received scholarships (n = 95). The mean score of parental attachment was 63.46 (min-max = 38-79, SD = 7.81). The average number of close friends was 5.25 (min-max = 0-100, SD = 6.41), and 5.17% (n = 43) of participants lived alone. The average score for relationship with friends/seniors/juniors/staff was 4.35 (min-max = 0-9, SD = 2.13), and the average score for ATSPPH-SF was 18 (min-max = 6-29, SD = 3.8) (Table 1).

The study received ethical approval from the Human Research Ethics Committee, Faculty of Medicine Ramathibodi Hospital, Mahidol University. The authors confirm that all the methods were carried out in accordance with relevant guidelines and regulations.

### Data collection

Data were collected via online questionnaire between July and September 2022. Participants were recruited through the heads of each year in the four bachelor's programs, who provided a link to an informed consent document and the online questionnaire to their classmates. All participants were informed of the research objectives and their right to voluntary participation before completing the questionnaire.

### Research instrument

The research instrument utilized in this study comprised a set of psychometric instruments designed to assess various aspects of the participants' mental health, rates of seeking mental health care, and related factors. The questionnaires included the following components:

Demographic data questionnaire A demographic data questionnaire containing 9 questions about participants’ gender, age, study programs, college years (preclinical/clinical), grade point average (GPA), scholarship status (received/not received), living situation (alone/with family or friends), socioeconomic status (had financial burden or not), and number of close friends. Participants were asked to select from options for most items but were required to fill in a blank space to provide their age and number of close friends.

**Table 1:** Descriptive analysis of demographic data and differences between programs (n= 832)

Demographics		Total (n = 832) n (%)/M (SD)	Medicine (n =330, 39.66%)**^†^** n (%)/M (SD)	Nursing (n =298, 35.82%)**^†^** n (%)/M (SD)	CSD (n = 98, 11.78%)**^†^** n (%)/M (SD)	Paramedicine (n =106, 12.74%)**^†^** n (%)/M (SD)	χ^2^/F	p
Gender	Male	152 (18.27)	114 (34.55)	8 (2.68)	11 (11.22)	19 (17.92)	121.30	< 0.001^***^
	Female	600 (72.10)	181 (54.85)	269 (90.27)	76 (77.55)	74 (69.87)		
	LGBTQ	80 (9.62)	35 (10.61)	21 (7.05)	11 (11.22)	13 (12.26)		
Age^‡, **¶**^		19.97 (1.74)	20.00 (1.70)	19.85 (1.20)	19.82 (1.22)	20.20 (3.04)	2.92	0.404
College year	Pre-clinic	438 (52.60)	208 (63.03)	149 (50.00)	47 (48.96)	34 (32.08)	33.97	< 0.001^***^
	Clinic	394 (47.40)	122 (36.97)	149 (50.00)	51 (52.04)	72 (67.92)		
GPA	≥ 3.50	447 (53.73)	210 (63.64)	120 (40.27)	48 (49.98)	69 (65.09)	41.63	< 0.001^***^
	≤ 3.49	384 (46.27)	119 (36.36)	178 (59.73)	50 (50.02)	37 (34.91)		
Received a scholarship	Yes	95 (11.42)	24 (7.27)	28 (9.40)	14 (14.29)	29 (27.36)	34.24	< 0.001^***^
	No	737 (88.58)	306 (92.73)	270 (90.60)	84 (85.71)	77 (72.64)		
Living	Alone	43 (5.17)	21 (6.36)	10 (3.34)	10 (10.20)	2 (1.89)	10.36	0.016^*^
	With family/friends	789 (94.83)	309 (93.64)	288 (96.64)	88 (89.80)	104 (98.11)		
Financially burdened	Yes	91 (10.94)	12 (3.64)	49 (16.44)	10 (10.20)	20 (18.87)	34.23	< 0.001^***^
	No	741 (89.06)	318 (96.46)	249 (83.56)	88 (89.80)	86 (81.13)		
Number of close friends**^‡^**^, **¶**^		5.25 (6.41)	6.11 (5.71)	4.26 (2.80)	4.35 (3.74)	6.23 (13.47)	31.98	< 0.001^***^
Relationship with friends/senior and junior/staffs score**^‡^**^, ¶^		4.35 (2.13)	4.10 (2.12)	4.48 (2.22)	4.83 (1.83)	4.29 (2.16)	11.44	0.010^*^
Parental attachment score**^‡^**^, **§**^		56.71 (4.31)	56.74 (4.49)	56.47 (4.24)	56.27 (4.16)	56.93 (4.11)	0.44	0.724
Any mental health problems		389 (46.80)	143 (43.33)	146 (48.99)	52 (53.06)	48 (45.28)	3.01	0.283
Self-harming thoughts and behaviors		303 (36.42)	115 (34.85)	106 (35.57)	42 (42.86)	40 (37.74)	2.27	0.517
Suicidal ideation (often-always)		130 (15.63)	50 (15.15)	52 (17.45)	15 (15.31)	13 (12.26)	1.72	0.632
Self-harm (often-always)		74 (8.89)	29 (8.79)	27 (9.06)	4 (4.08)	14 (13.21)	5.25	0.154
Suicidal plans		158 (18.99)	63 (19.09)	60 (20.13)	17 (17.35)	18 (16.98)	0.71	0.872
Attempted suicide**^#^**		50 (6.01)	17 (5.15)	22 (7.38)	5 (5.10)	6 (5.66)	9.95	0.620
Anxiety		162 (19.47)	51 (15.45)	77 (25.84)	19 (19.39)	15 (14.15)	13.02	0.005^**^
Depression		67 (8.05)	28 (8.48)	27 (9.06)	5 (5.10)	7 (6.60)	1.95	0.584
Seeking mental health care								
Formal mental health care		109 (13.10)	65 (19.70)	28 (9.40)	9 (9.18)	7 (6.60)	21.46	< 0.001^***^
From within the program/university		64 (7.69)	44 (13.33)	15 (5.03)	3 (3.06)	2 (1.89)	25.75	< 0.001^***^
From outside the university		63 (7.57)	32 (9.70)	17 (5.70)	8 (8.16)	6 (5.66)	4.22	0.239
Informal mental health care^# ^		816 (98.08)	322 (97.58)	295 (99.00)	96 (98)	103 (97.17)	2.42	0.490
From friends		306 (92.73)	292 (97.99)	94 (95.92)	100 (94.34)	9.74	0.021	0.021^*^
From family		281 (85.15)	252 (84.56)	89 (90.82)	90 (84.91)	2.52	0.473	0.473
From staff		115 (34.85)	125 (41.95)	40 (40.82)	44 (41.51)	3.90	0.273	0.273
ATSPPH-SF**^‡^**^, **§**^		17.99 (3.79)	18.06 (3.94)	18.17 (3.58)	18.24 (3.61)	17.03 (3.99)	2.65	0.485
Perceived mental health service information								
Yes		661 (79.45)	263 (79.70)	257 (86.24)	57 (58.16)	84 (79.25)	35.62	< 0.001^***^
No/Not sure		171 (20.55)	67 (20.30)	41 (13.76)	41 (41.84)	22 (20.75)		
Attitude towards mental health services**^‡^**^, **¶**^		11.60 (2.30)	11.28 (2.25)	12.07 (2.04)	11.12 (2.02)	11.69 (2.29)	24.20	< 0.001^***^

Note: † = percentage compared to the total number of samples, ‡ = mean (SD), ¶ = Kruskal Wallis Test, § = ANOVA test,  #  =  test with Fisher’s Exact Test / Likelihood Ratio as appropriate for the information; The symbols *, **, and *** denote p-values significant at <.05, <.01, and <.001, respectively; Abbreviations:  SD = standard deviation; LGBTQ = lesbian, gay, bisexual, transgender, and queer; GPA = grade point average; CSD = communication sciences and disorders program, NSSI = non-suicidal self-injury, ATSPPH-SF = attitudes toward seeking professional psychological help scale-short form

### Rates of seeking mental health care assessment

The assessment of the rates of seeking mental health care consisted of a five-item questionnaire developed by the researchers. For informal mental health care, there were 3 items that asked participants if they had ever sought counseling or help from family, friends, or program staff. For formal mental health care, there were 2 items that asked if participants had ever sought counseling or help from psychiatrists or psychologists at hospitals/clinics/counseling centers within the programs/university or from outside. Each item had 4 response options (never, sometimes, often, and always), and participants who chose “sometimes”, “often”, or “always” were categorized as having sought help from formal or informal mental health care. The internal consistency of the questionnaire utilized in this study was found to be unacceptable with a Cronbach's alpha coefficient of 0.29. However, it is important to note that this questionnaire measures how often participants seek mental health care, and seeking help from others around them may vary from individual to individual. It is common for individuals to seek help from those who are close to them, such as family and friends, more than from individuals who are less familiar, such as teachers or mental health professionals.^17^ Furthermore, the small number of items in this questionnaire may have affected the Cronbach's alpha score, as the coefficient is known to be sensitive to the number of items. Nevertheless, it is worth noting that with a small number of items, a low Cronbach's alpha may still be considered a valid questionnaire.^18^^, ^^19^

### Mental health problems assessments

Self-harm behaviors and suicidal ideation were assessed using the self-harm and suicidal ideation part of the Impulse, Self-harm and Suicide Ideation Questionnaire for Adolescents (ISSIQ-A), which  developed by Castilho, P., Carvalho, C. B. & Pinto‐Gouveia.^20^ The questionnaire consists of 13 items that assess self-harm behaviors, including non-suicidal self-injury (NSSI) (8 items) and health risk behaviors (5 items), as well as 3 items that assess suicide ideation. Each item is rated on a 4-point Likert scale (never, sometimes, often, and always), and participants who select “often” or “always” on any item are classified as having those problems. The internal consistency of the overall questionnaire in this study was acceptable (Alpha = 0.77), while the internal consistency for the self-harm and suicide ideation subscales were questionable (Alpha = 0.69) and acceptable (Alpha = 0.79), respectively. The questionnaire's indexes of Item Objective Congruence (IOC) were 0.89, as determined by four experienced psychiatrists who treat patients with NSSI or suicide.

Suicidal plan and attempt were assessed using the Thai Youth Risk Behavior Survey (YRBS), which is a translated version of Youth Risk Behavior Surveillance System (YRBSS) questionnaire developed by the Centers for Disease Control and Prevention (CDC) by Ruangkanjanaset S..^21^Two questions related to suicidal behavior were selected to used in this study. The first questions asked if participants had made a plan for attempting suicide, with options of yes or no. The second question asked how many times they had attempted suicide in the last 12 months, with response options ranging from 0 to more than 6 times. Participants who selected any option from 1 to more than 6 times were classified as having had a suicidal attempt. However, it is worth noting that the questionnaire demonstrated poor reliability in this study (Alpha = 0.52). This may be due to differences between suicidal plan and attempt, where some individuals who had suicidal plans did not have a history of suicidal attempts, while others who had attempted suicide may not have had previous suicidal plans.^22^ Additionally, the small number of items included in the questionnaire may have contributed to the low Cronbach's alpha value. Nevertheless, it is important to note that a low Cronbach's alpha value does not necessarily imply that a questionnaire is invalid, especially when the number of items is small.^18^^, ^^19^ Moreover, this questionnaire is internationally accepted and widely used in various research studies.^23^^,^^24^

Depression and anxiety were assessed using the Hospital Anxiety and Depression Scale Thai version (Thai HADS), translated by Nilchaikovit T. and colleagues.^25^ This self-report questionnaire comprises 14 items, with 7 items each for depression and anxiety symptoms. Responses are rated on a 4-point Likert scale ranging from 0 to 3, with higher scores indicating more severe depression and anxiety symptoms. Total scores on the questionnaire can be interpreted as follows: 0-10 as non-cases, and 11-21 as cases of anxiety or depression. The questionnaire showed good internal consistency for both anxiety and depression subscales (Alpha = 0.86 and 0.83, respectively), indicating good reliability of the items in measuring the constructs.

### Attachment to parent assessment (IPPA-R)

Measured using the parental part of the Inventory of Parent and Peer Attachment-Revised (IPPA-R). This questionnaire, developed by Gullone E. and Robinson K.^26^ and translated into Thai by Lakthong A,^27^ consists of 28 items assessing attitude toward their parents, which are divided into three subscales: trust (10 items), communication (10 items), and alienation (8 items). Responses are scored on a 5-point Likert scale, ranging from 1 (almost never or never true) to 5 (almost always or always true). The total score is the sum of all items and ranges from 28 to 140, with higher scores indicating greater attachment to parents. The questionnaire demonstrated good reliability in this study (Alpha = 0.88).

### Attitudes Toward Seeking Professional Psychological Assessment (ATSPPH-SF)

The Thai version of Attitudes Toward Seeking Professional Psychological Help Scale-Short Form (ATSPPH-SF) by Fischer and Farina^28^ was used to measure attitudes toward seeking mental health care from professionals. The questionnaire was translated into Thai by the researchers and back translated by two experts who are proficient in both English and Thai language. The measure consisted of 10 self-rated items. Responses are rated on a 4-point Likert scale ranging from 0 to 3, with higher scores indicating more higher attitudes toward seeking mental health care from professionals. The contents validity of this measurement was assessed by three experts using a content validity index (CVI), which yield a result of 1. The linguistic accuracy of the questionnaire was also evaluated by administering it to 10 students. The questionnaire demonstrated good overall internal consistency (Alpha = 0.84).^28^

### Attitude and satisfaction with mental health services

The authors developed a 6-item self-rated questionnaire to measure attitude and satisfaction with mental health services. The items were developed based on a review of previous studies related to attitudes and satisfaction with mental health services.^29^^, ^^30^ The first item asked about the availability of mental health services provided by their programs, faculty, or university. The remaining items asked about attitudes and satisfactions related to mental health services provided by the college, such as “Do you agree that program/faculty/university mental health services are private? Do you agree that program/faculty/university mental health services maintain confidentiality?”. Responses are rated on a 4-point Likert scale ranging from strongly disagree to strongly agree, with higher scores indicating higher satisfaction with mental health services. To assess the content validity of this measurement, three experts to assess the content validity of this measurement, with a result of 1. The questionnaire was also evaluated for linguistic accuracy by administering it to 10 students. The questionnaire demonstrated good internal consistency (Alpha = 0.82).

### Statistical analysis

All data analyses were performed by means of SPSS version 26.0. The descriptive statistics were used to analyze demographic data, mental health problems, parental attachment, attitudes toward seeking professional psychological help, and attitude and satisfaction with mental health services. The frequency, percentage, mean, and standard deviation were calculated for each variable. To investigate the factors associated with mental health problems and seeking mental health care, Pearson’s chi-square and t-test were used. Binary logistic regression was used to analyze factors that predict seeking mental health care. In this analysis, only participants who reported self-harm, suicidal attempt, or ideation, and those who are cases of anxiety or depression were included. Statistical significance was set at 5% (p = 0.05; two-sided) throughout the analysis.

## Results

### Prevalence of mental health problems and rates of seeking mental health care

From Table 1, The result indicated that 46.80% (n = 389) of the participants reported experiencing any mental health problems. Of these, 36.42% (n = 303) reported self-harm behaviors or suicidal ideation, 18.99% (n = 158) who reported suicidal plan and 15.63% (n = 130) who reported suicidal ideation. Additionally, it was found that 19.47% (n = 162) and 8.05% (n = 67) of the participants had anxiety or depression, respectively.

Among the sample with mental health problems (n = 389), 97.94% (n = 381) reported seeking informal health care, while only 16.97 % (n = 66) had sought formal health care from professionals. However, there were a small number of participants (n = 8, 2.06%) who reported not seeking help from any source (Table 2). Furthermore, it was found that participants with anxiety were more likely to seek formal mental health care from sources outside of the university compared to those with other type of mental health problems (χ^2^_(1, N = 389)_ = 7.98, p = 0.005), while participants with depression were more likely to seek help from their families (χ^2^_(1, N = 389)_ = 11.32, p = 0.001) and professionals than those with other mental health problems (χ^2^_(1, N = 389)_ = 9.54, p = 0.002).

### Factors associated with seeking mental health care among students with self-harm thoughts and behaviors Anxiety and Depression

According to Table 3, Among individuals with mental health problems, LGBTQ and female participants were more likely to seek informal mental health care than male participants (χ^2^_ (2, N = 389)_ = 8.50, p = 0.014). Additionally, our data indicated that those who were LGBTQ (χ^2^_ (2, N = 389)_, p = 0.002), studied in the medical program (χ^2^_ (3,N = 389)_  = 11.13, p = 0.011), did not receive scholarships (χ^2^_ (1, N = 389)_ = 6.94, p = 0.008), lived alone (Fisher’s exact test p = 0.008), had lower scores on measures of relationships with friends/seniors/juniors/staff (t _(387)_ = 2.08,  p = 0.037), and had lower parental attachment scores (t _(387)_ = 2.51, p = 0.012) were more likely to seek formal mental health care.

### Factors affecting seeking mental health care of students with mental health problems

According to Table 4, female and LGBTQ participants were 2.63 (CI = 1.08 - 6.43, p = 0.034) and 4.26 (CI = 1.36 - 13.37, p = 0.013) times more likely to seek formal mental health care than male participants, respectively. Additionally, individuals studying in CSD and nursing programs were found to be 0.30 (CI = 0.10 - 0.92, p = 0.035) and 0.28 (CI = 0.14 - 0.58, p = 0.001) times less likely to seek formal mental health care than those studying in a medicine program, respectively. Furthermore, participants with a one-point increase in their attitude towards help seeking were 1.10 times more likely to seek mental health care (CI = 1.02 - 0.19, p = 0.010). Lastly, those who received a scholarship were 3.30 times more likely to seek formal mental health care than those who did not (CI = 1.35 - 8.06, p = 0.009)

## Discussion

The present study found a high prevalence of mental health problems among health-related students, with a majority having sought counseling from friends and family, but only a small number seeking care from mental health experts. Furthermore, individuals who identified themselves as females or LGBT, studied in the medical program, had received scholarships, and had a positive attitude toward seeking help from experts were more likely to seek formal mental health care.

This study indicated that the prevalence of suicidal ideation was 15.63%, which was higher than those reported in prior Thai and international studies (6.4 - 12.2 %) of general college students.^31^^-^^33^ Moreover the prevalence of suicidal plans and attempted suicide (18.99 and 6.01% respectively) were also higher than the results of systematic literature reviews among general college students in which the prevalence of suicide planning was 15.5-16.1%^34^^, ^^35^ and suicide attempts was 3-4.37%.^34^^, ^^36^ Similarly, the prevalence of anxiety in this study, 43.75%, was found to be the higher than reported in previous systematic review studies, which were only 31-32%.^37^^,^^38^ One possible explanation for higher rates of these mental health problems among health-related college students may be the highly competitive study environments and the numerous demands they face, such as lack of rest and leisure activities.^39^^,^^40^Additionally, as health care personnel have easy access to self-prescribing, they may have more methods of suicide at their disposal than students in other fields.^41^ Furthermore, the data were collected during the Covid-19 pandemic period may also added more stress, and increased mental health issues for students using online learning platforms.^42^^,^^43^

On the other hand, the study found a lower-rate of self-harm (8.89%) compared to prior research that reported a prevalence of 17-20%.^44^^-^^46^ This mat due to the relatively high cut-off point for self-harm behavior used in this study, “often” or “all the time”, which is higher than prior studies that have used a behavioral cut-off point of self-harm behavior in the past 12 months regardless of frequency of behavior^44^ or only one or more self-harming behaviors.^44^^-^^46^ Additionally, the prevalence of depression in this study (21.51%) was found to be somewhat lower than in previous systematic review studies which was 24-36.5%.^13^^, ^^37^^, ^^38^ This may be due to differences in measurement tools and cut-off points for depression.

**Table 2:** The prevalence of seeking mental health care among individuals with mental health problems (n= 389)

Variable			Any mental health problems (n = 389, 46.80%)**^†^**n (%)	Self-harming thoughts and behaviors (n = 303, 36.42%)**^†^** n (%)		χ^2^	p	Anxiety (n = 162, 17.3%)**^†^** n (%)		χ^2^	p	Depression (n = 67, 7.2%)**^†^** n (%)		χ^2^	p
				Yes	No			Yes	No			Yes	No		
Seeking mental health care		Y	381(97.94)	297(98.02)	84(97.67)	N/A	1.000	158(97.53)	223(98.24)	N/A	0.724	66(98.51)	315(97.83)	N/A	1.000
		N	8(2.06)	6(1.98)	2(2.33)			4(2.47)	4(1.76)			1(1.49)	7(2.17)		
Seeking informal mental health care		Y	381(97.94)	297(98.02)	84(97.67)	N/A	1.000	158(97.53)	223(98.24)	N/A	0.724	66(98.51)	315(97.83)	N/A	1.000
		N	8(2.06)	6(1.98)	2(2.33)			4(2.47)	4(1.76)			1(1.49)	7(2.17)		
	Friends	Y	370(95.12)	289(95.38)	81(94.19)	N/A	0.583	154(95.06)	216(95.15)	0.00	0.967	64(95.52)	306(95.03)	N/A	1.000
		N	81(4.88)	14(4.62)	5(5.81)			8(4.94)	11(4.85)			3(4.48)	16(4.97)		
	Family	Y	322(82.78)	255(84.16)	67(77.91)	1.84	0.175	129(79.63)	193(85.02)	1.93	0.165	46(68.66)	276(85.71)	11.32	0.001^**^
		N	67(17.22)	48(15.84)	19(22.09)			33(20.37)	34(14.98)			21(31.34)	46(14.29)		
	Staff	Y	144(37.02)	106(34.98)	38(44.19)	2.43	0.119	68(42.0)	76(33.5)	2.93	0.087	24(35.82)	120(37.27)	N/A	1.000
		N	245(62.98)	197(65.02)	48(55.81)			94(58.0)	151(66.5)			43(64.18)	202(62.73)		
Seeking formal mental health care		Y	66(16.97)	52(17.16)	14(16.28)	0.04	0.847	34(20.99)	32(14.10)	3.19	0.074	20(29.85)	46(14.29)	9.54	0.002^**^
		N	323(83.03)	251(82.84)	72(83.72)			128(79.01)	195(85.90)			47(70.15)	276(85.71)		
	Within the programs /university	Y	40(10.28)	33(10.89)	7(8.14)	0.55	0.458	21(12.96)	19(8.37)	2.16	0.141	12(17.91)	28(8.70)	5.11	0.024^*^
		N	349(89.72)	270(89.11)	79(91.86)			141(87.04)	208(91.63)			55(82.09)	294(91.30)		
	Outside the university	Y	40(10.28)	30(9.90)	10(11.63)	0.22	0.642	25(15.43)	15(6.61)	7.98	0.005^**^	13(19.40)	27(8.39)	7.30	0.007^**^
		N	349(89.72)	273(90.10)	76(88.37)			137(84.57)	212(93.39)			54(80.60)	295(91.61)		

Note: † = percentage compared to the total number of samples; The symbols *, **, and *** denote p-values significant at <.05, <.01, and <.001, respectively; Abbreviations: Y= seeking mental health care; N = not seeking mental health care

**Table 3:** The factors associated with seeking mental health care among individuals with mental health problems (n= 389)

Variable		Seeking informal mental health care		χ^2^ or t	p	Seeking formal mental health care		χ^2^ or t	p
		n (%)/M (SD)				n (%)/M (SD)			
		yes	no			yes	no		
Gender^†^	Male	67 (93.06)	5 (6.94)	8.50	0.014^*^	8 (11.11)	64 (88.89)	12.69	0.002^**^
	Female	276 (98.92)	3(1.08)			44 (15.77)	235 (84.23)		
	LGBTQ	38 (100.00)	0 (0.00)			14 (36.84)	24 (36.16)		
Age^‡^		20.04 (1.72)	20.38 (1.1)	0.54	0.590	20.28 (1.5)	20.01 (1.7)	-1.07	0.282
Study programs	Medicine	140 (97.90)	3 (2.10)	1.04	0.790	36 (25.17)	107 (74.83)	11.13	0.011^*^
	CSD	50 (96.15)	2 (3.85)			5 (9.62)	47 (90.38)		
	Nursing	144 (98.63)	2 (1.37)			19 (13.01)	127 (86.99)		
	Paramedicine	47 (97.92)	1 (2.08)			6 (12.50)	42 (87.50)		
ATSPPH-SF^‡^		18.23 (4.06)	17 (4.96)	-0.84	0.401	19.11 (4.55)	18.02 (3.95)	-1.98	0.048^*^
Received a scholarship	Yes	37 (100.00)	0(0.00)	N/A	1.00^†^	12 (32.43)	25 (67.57)	6.94	0.008^**^
	No	344 (97.73)	8 (2.27)			54 (15.34)	298 (84.66)		
Living	Alone	359(98.09)	22(95.65)	N/A	0.389^†^	57 (15.57)	309(84.43)	N/A	0.008^**,†^
	Family/friends	7(1.91)	1(4.35)			9(39.13)	14(60.87)		
Relationship with friends/senior and junior/staffs^‡^		4.02 (2.14)	3.63 (2.13)	0.76	0.604	3.52 (2.12)	4.12 (2.12)	2.08	0.037^*^
Number of close friends		4.87 (6.54)	3.25 (3.32)	-0.69	0.485	4.67 (4.42)	4.88 (6.84)	0.23	0.812
Parental attachment^‡^		61.02 (8.31)	58.63 (9.41)	-0.80	0.423	58.64 (7.45)	61.44 (8.43)	2.51	0.012^*^
Financially burdened	Yes	56 (96.55)	2 (3.45)	N/A	0.340^†^	11 (18.97)	47 (81.03)	0.19	0.660
	No	325 (98.19)	6 (1.81)			55 (16.62)	276 (83.38)		
Perceived mental health service information	Yes	292 (97.66)	7 (2.34)	N/A	0.688^†^	54 (18.06)	245 (81.94)	1.09	0.295
	No	89 (98.89)	1 (1.11)			12 (13.33)	78 (86.67)		
Attitude towards mental health services^‡^		11.44 (2.30)	12.75 (2.25)	1.59	0.112	11.21 (2.30)	11.51 (2.33)	0.96	0.334

Note: † = test with Fisher’s Exact Test / Likelihood Ratio as appropriate for the information, ‡ = mean (SD) with t-test analysis; The symbols *, **, and *** denote p-values significant at <.05, <.01, and <.001, respectively. ; Abbreviations: M = mean; SD= standard deviation; LGBTQ = lesbian gay bisexual transgender queer; CSD = communication sciences and disorders program; ATSPPH-SF = attitudes toward seeking professional psychological help scale-short form

The study also found that most participants with mental health problems (97.94%) had consulted with people they were close to. This result was higher than previous studies which showed that 48% of the students had been to be counselling.^14^ Most of the students sought help from those they are close to, and they were more likely to solve problem independently than seeking profession care.^47^^,^^48^   These were correlated with our finding which found only 16.97% of those who had mental health problems sought professional help. However, this rate was higher than that of students and general population admissions to mental health care, which was found to be only 4.1-6%.^8^^,^^49^ One possible explanation for this difference is that the majority of our participants had received training on mental health and treatment options during their first year of study. This training likely helped to increase students’ understanding of mental health and positively influenced their attitudes towards seeking mental health services,^50^ which was found to be one of the factors associated with seeking mental health care in this study.

However, the study showed 8 participants (2.06%) with mental health problems never received counseling. In a previous study, it was addressed that 49% of medical students did not want to work with those with mental issues and perceived that asking for help meant that a person was incapable of dealing with himself/herself or was unable to manage other things well.^51^^,^^52^ In addition, people with depression tend to isolate, which is why they do not seek help and might eventually lead to suicide attempts.^53^

The study found that female and LGBTQ participants were more likely to seek formal mental health care than male participants, which was consistent with previous studies. A previous research has shown that females are more interested in mental health information, recommended mental health services to their friends, and seek help from mental health institutions than males.^54^ Additionally, some LGBTQ students have reported positive experience with using health services, especially those that sensitive to gender identity or met with professionals with high experience and knowledge in taking care of LGBTQ individuals. However, there are still some LGBTQ participants who are hesitant to seek mental health care due to concerns about exposure of their gender identity or sexual orientation.^55^ Therefore, it is important that mental health professionals are well-trained and have a good understanding of how to provide care for the LGBTQ population. Besides, males may be more likely to be ashamed to seek counseling and toperceive seeking help as a sign of weakness.^30^^,^^56^ Moreover, males tend to use other stress management methods such as alcohol or drug abuse, which can negatively impact their health in the long term.^57^

In this study, different programs were found to be associated with different rates of seeking mental health care. This may be due to differences in mental health problems and attitudes towards counseling among students in each program.^47^^, ^^52^^, ^^58^ These factors were also found to differ among the study programs in this study. Furthermore, students who received scholarships were found to be more likely to seek formal mental health care. This may be because scholarship recipients often go through an interview process and are assessed by a scholarship reviewers, who may identify students’ stress and advise them to seek counseling.

A positive attitude towards seeking help was also found to be a factor that increased the likelihood of seeking psychological help. Health-related students tend to have knowledge and understanding of mental health problems, which can reduce feelings of anxiety or stigmatization and make them more willing to seek mental health care.^59^^,^^60^ This is consistent with the findings of this study, which found that the mean score of the ATSPPH-SF in health-related students (mean =19.11) was higher than in previous studies in the general European population (mean = 17.4)^61^ and the Chinese population (mean = 18.13)^62^. Previous studies have found that those who have attended mental health training programs or received quality education on mental health information are more likely to have accurate knowledge and information on psychiatric disorders and reduce negative attitudes towards these disorders. This knowledge also helps improve decision making in seeking mental health care, both formally and informally.^63^^, ^^64^,^65^

In this study, the results show that parental attachment was not associated with seeking formal mental health care. This contradicts to previous studies which found that families who are aware of their children's problems, perceived and saw the impact of mental health problems on their children, had a good family’s support network were more likely to seek help and access mental health services than family without the aforementioned characteristics.^11^^, ^^66^ However, when we examined the relationship between family ties and seeking psychological support from family members, we found that individuals with mental health problems and good relationships with their parents were more likely to seek psychological support from their parents than those with poor parental attachment. Additionally, our study found that attitudes towards mental health services were not related to seeking formal mental health care. This may be due to the fact that most of the participants in our study had relatively positive attitudes towards mental health services, resulting in a lack of statistically significant correlation.

### Limitations

This study contributes to the understanding of seeking mental health care among health-related students who have mental health problems. However, the study still has several limitations. Firstly, the cross-sectional design of the study does not allow for conclusions regarding causality. Secondly, the sample is limited to students from only one university, which may not be generalized to students from other universities or fields. Future research should consider including a more diverse sample to enhance the generalizability of the results. Thirdly, it is worth noting that the internal consistency of some of the questionnaire sets was low, possibly due to the small number of items included. While low Cronbach's alpha values may be indicative of poor reliability, a small number of items does not necessarily mean that the questionnaire is invalid or unusable.^18^^, ^^19^

**Table 4:** Regress analysis of factors affecting seeking formal mental health care among students with mental health problems (n = 389)

Factor		seeking formal mental health care		
		OR	(95% CI)	p
Gender^†^	Female	2.63	(1.08-6.43)	0.034^*^
	LGBTQ	4.26	(1.36-13.37)	0.013^*^
Age		1.00	(0.83-1.21)	0.976
Study programs^‡^	CSD	0.30	(0.10-0.92)	0.035^*^
	Nursing	0.28	(0.14-0.58)	0.001^*^
	Paramedicine	0.38	(0.14-1.05)	0.062
Receiving a scholarship^¶^		3.30	(1.35-8.06)	0.009^**^
Living^§^		2.68	(0.89-8.07)	0.080
ATSPPH-SF		1.10	(1.02-0.19)	0.010^*^
Relationship with friends/senior and junior/staffs		0.92	(0.79-1.08)	0.307
Parental attachment		0.97	(0.93-1.01)	0.154
Self-harming thoughts and behaviors		1.58	(0.70-3.58)	0.273
Anxiety		1.62	(0.79-3.33)	0.190
Depression		1.93	(0.89-.4.19)	0.096
Perceived services information^#^		2.07	(0.94-4.67)	0.080
Attitude towards mental health services		0.04	(0.89-1.16)	0.824

Note: † reference = male, ‡ reference = medical program, ¶ reference = did not receive a scholarship, § reference = living with family/friends, # reference = did not perceived service information; The symbols *, **, and *** denote p-values significant at < .05, <.01, and <.001, respectively; Abbreviations: OR = odd ratio; CI = confidence interval; LGBTQ = lesbian gay bisexual transgender queer; CSD = communication sciences and disorders program; ATSPPH-SF = attitudes toward seeking professional psychological help scale-short form

However, in subsequent studies, it would be advisable to increase the number of items to improve the questionnaire's internal consistency. Fourthly, the self-report nature of the data may have introduced response bias or social desirability bias as participants may have underreported or overreported their symptoms or health-seeking behaviors, which can potentially affect the validity of the results. Finally, there may be other factors related to seeking mental health care, such as satisfaction level with mental health care services and support from society that are not addressed in this study and should be considered in future research.

## Conclusions

Mental health problems are a significant concern among college students, as evidenced by the high prevalence rates reported in this study. Unfortunately, a relatively small proportion of students seek help for these issues from professionals. This study underscores the need for greater awareness of mental health issues among college personnel responsible for student well-being. Furthermore, promoting positive and accurate attitudes toward mental health problems through education, such as mental health pamphlets, and providing basic counselling skills for college students or faculty staffs, as well as improving the quality of mental health services, may reduce mental health problems and increase access to mental help support among college students. There are several recommendations for future research in this area. Firstly, further investigation into the factors that influence help-seeking behaviors among college students is necessary to identify effective strategies to promote access to mental health services. Secondly, additional research is needed to evaluate the effectiveness of various interventions aimed at improving mental health literacy and reducing the stigma surrounding mental health issues. Thirdly, future studies should investigate the potential benefits of integrating mental health education and basic counselling skills into college curricula.

### Acknowledgments

We thank Wanlop Atsariyasing, Nida Limsuwan, and Thanavadee Prachason for assessing the Content Validity Index (CVI) of the questionnaire. Thank Sudawan Jullagate and Pattarabhorn Wisajun for coordinate with ethical committee and assist with statistical analysis. Lastly, we would also like to thank heads of each year in the four bachelor's programs who helped coordinate with classmates in collecting data for this research.

### Conflict of Interest

The authors declare that they have no conflict of interest.
